# Is combinatorial chemistry on the right track for drug discovery?

**DOI:** 10.1155/S1463924603000099

**Published:** 2003

**Authors:** Richard A. Wildonger, Tracy L. Deegan, John W. Lee

**Affiliations:** Chemical Technology Section Enabling Science and Technology Department AstraZeneca R&D Boston Waltham MA 02451 USA

## Abstract

Critical to the effective implementation of high throughput methods of synthesis is the necessity for a significant supporting level of automation. There are a number of critical issues associated with the successful introduction, and supporting role, of automation of small molecule chemical synthesis. Clearly there are needs for automation to increase drug candidate synthesis throughput. Automation of repetitive and laborious tasks associated with the synthesis process can release skilled chemists to apply their talents to the more challenging investigational aspects of developing new synthetic protocols. This provides continuity in the compound supply pipeline and ensures an optimal use of the automated platform for compound production. The very high fidelity of performing repetitive processes that can be managed through automation also removes some of the limitations and errors associated with more fallible human operators. This can include very difficult tasks associated with tracking data, and general information and inventory management. Taken collectively, these attributes associated with automation can lead to greater efficiencies, throughputs and improved allocation of human resources with concomitant reductions in costs associated with current day and future drug discovery. In our library development/synthesis paradigm, we feel that automation support must be invoked early in the process and that this automation support must continue throughout the project.


Journal of Automated Methods & Management in Chemistry
Vol. 25, No. 3 (May–June 2003) pp. 57–61

Taylor & Francis

Taylor & Francis Group

### Is combinatorial chemistry on the right track for drug discovery?†

**Richard A. Wildonger, Tracy L. Deegan and John W. Lee**

*Chemical Technology Section, Enabling Science and Technology Department, AstraZeneca R&D Boston, Waltham, MA 02451, USA*

*Critical to the effective implementation of high throughput methods of synthesis is the necessity for a significant supporting level of automation. There are a number of critical issues associated with the successful introduction, and supporting role, of automation of small molecule chemical synthesis. Clearly there are needs for automation to increase drug candidate synthesis throughput. Automation of repetitive and laborious tasks associated with the synthesis process can release skilled chemists to apply their talents to the more challenging investigational aspects of developing new synthetic protocols. This provides continuity in the compound supply pipeline and ensures an optimal use of the automated platform for compound production. The very high fidelity of performing repetitive processes that can be managed through automation also removes some of the limitations and errors associated with more fallible human operators. This can include very difficult tasks associated with tracking data, and general information and inventory management. Taken collectively, these attributes associated with automation can lead to greater efficiencies, throughputs and improved allocation of human resources with concomitant reductions in costs associated with current day and future drug discovery. In our library development/synthesis paradigm, we feel that automation support must be invoked early in the process and that this automation support must continue throughout the project.*

### Introduction

High throughput synthesis, aided by combinatorial techniques, automated synthesis and parallel purification, has contributed a great deal to making the synthetic medicinal chemist more efficient. However, it is not as clear that these techniques alone have made drug discovery easier or more successful. Other tools have become available to aid in library design, while cheminformatics provide a rich base for knowledge-driven discovery. Structure-based design has been available for several decades, but has enjoyed a rebirth due to the increased sophistication of modeling software and the availability of many attractive target crystal structures.

The guiding principle of medicinal chemists is the acquisition of knowledge. The quandary is often in choosing which molecules to make. These should be chosen in a manner that maximizes the understanding of the relationship between chemical structure and biological activity. The compounds should be 'drug-like' in character and should effectively address ongoing hypotheses regarding the rules for improving activity in a given series [1–3].

### Are combinatorial chemistry tools advancing discovery?

There are inherent limitations in the available technologies. There are activation barriers to learning new systems, particularly if end-user intimacy is sacrificed. Medicinal chemists find themselves dependent on other technologies and may be uncomfortable in that role. As end-users they must judge the adequacy of the particular tool for the particular application. And in many cases will find that the new tools may be only partially enabling in accessing the molecules that they need. There are also potential traps if tools are misplaced. Several questions need to be addressed:

- Is the library design diverse and drug-like?
- Is the chemistry accessible through automation?
- Am I overloaded with data, much of which is irrelevant?
- Is there non-stochastic noise in the data causing me to miss something important?

Any or all of these factors can lead to the neutralization of enablement.

### Genome-based drug discovery

In principle, the human genome provides more than 100 000 potential targets. However, before embarking on genome-based drug discovery, we need to assess a number of important questions.

- How many are drugable?
- For what diseases?
- How do we choose intelligently?
- How do we validate those, which are chosen?
- How long will it take?
- What will it cost?

Unfortunately, many of these novel targets are extremely knowledge poor. Indeed, building a knowledge base on a novel target is a very large investment of both time and money.

### Large library approach

Figure 1 shows a split-pool combinatorial library comprised of over 2 million compounds [4]. This library of polycyclic shikimic acid analogues was elegantly synthesized with great attention to quality control.

*To whom correspondence should be addressed.
e-mail: richard.wildonger@astrazeneca.com
†This paper was initially presented at the ISLAR 2002 Conference and is reproduced here by kind permission of Zymark Corporation.

*Journal of Automated Methods & Management in Chemistry* ISSN 1463–9246 print/ISSN 1464–5068 online © 2003 Taylor & Francis Ltd
http://www.tandf.co.uk/journals
DOI: 10.1080/1463924032000121110

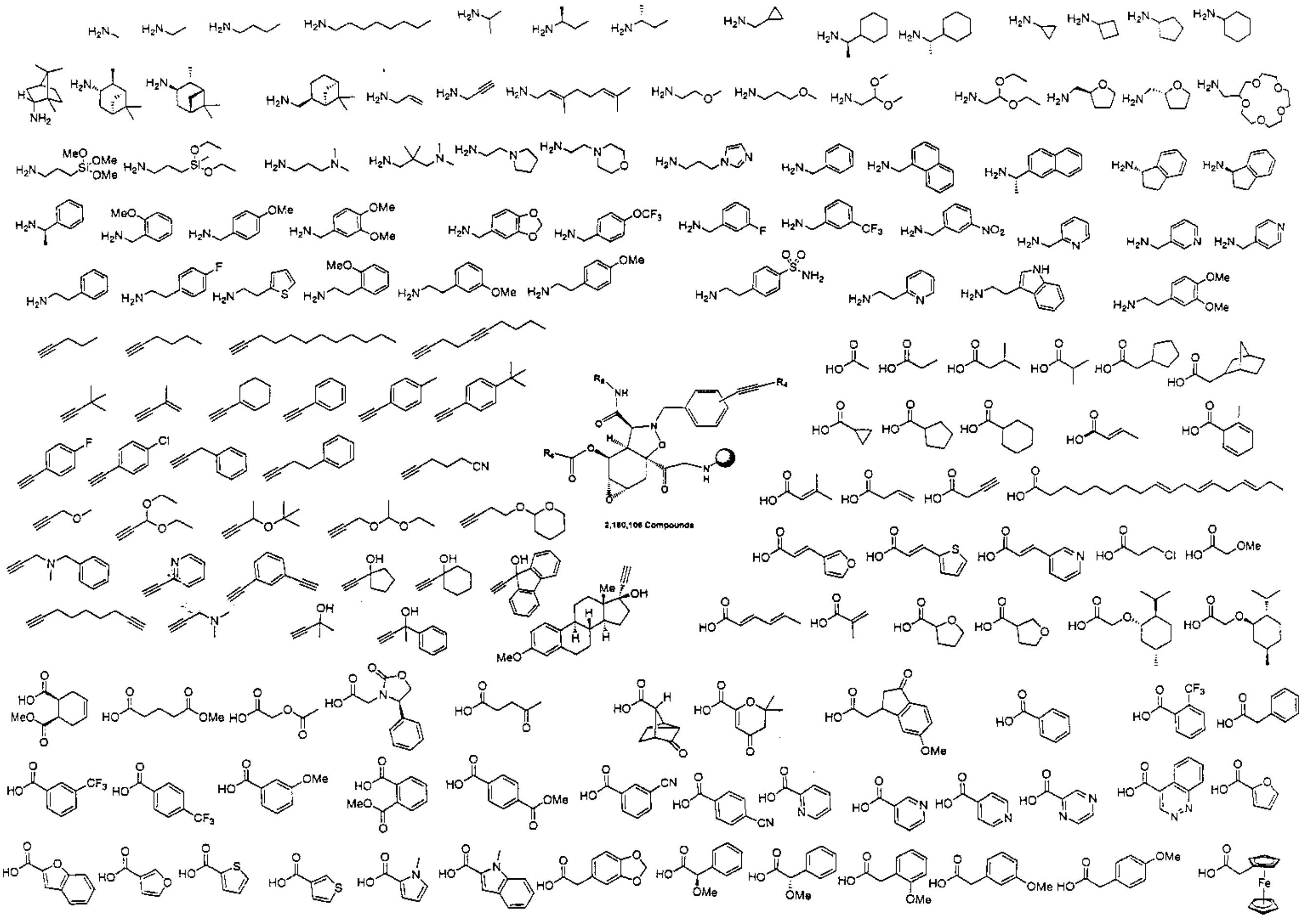

*Figure 1. Full matrix combinatorics using split-pool organic synthesis.*

See [4] for an excellent account of this elegant synthetic work. However, this library was screened in three separate assays. No activity was observed in the first two, and in the third assay the best activity was $EC_{50} = 50\,\mu M$ as an activator of the TGF-$\beta$-responsive mammalian reporter gene. This work highlights the dangers involved in screening mixtures of compounds. Unfortunately, only a very few biologically active small molecules are highly selective for their targets. Productive libraries are probably best built on known biologically active small molecules. The goal should be to identify compounds which do the following:

- Exhibit confirmed activity in at least one HTS assay.
- Have been filtered to remove objectionable functionality.
- Have passed selected 'drug-like' algorithms.
- Have passed a 'non-toxic' algorithm.

At this point enhancement of potency and selectivity for target identification can be achieved with a series of synthesize, test, design cycles.

### *Systematic synthesis development*

The development of synthetic strategies needs to be carefully systematized to maximize efficiency and chemistry reliability. This can best be accomplished by following a tightly defined series of developmental steps.

- Replicate model literature reactions.
- 'Parallelize' the development of solid phase and solution phase synthesis.
- Have a clear definition of experimental expectations:
  - Rigorous analysis using internal standards.
  - Run experiments in multiplicate.
- Formal experimental design and optimization:
  - Select variables.
  - Stage early on automated platform.
  - Iterate.
- Avoid restricting the options too early.
- Allow the chemistry to guide the use of appropriate automation rather than forcing the chemistry to work on an existing automated platform.

Overall, we expect automation to reproduce those actions faithfully, which are performed manually by chemists. This reliability should remain even in the face of complex syntheses. Automated platforms should not be unduly fettered by a complex learning curve. Of course, of necessity the automation must be priced to fit the research budget while at the same time remaining fit for purpose. We make every effort to use the appropriate-automated technique from the very beginnings of exploratory chemistry, chemistry development, library

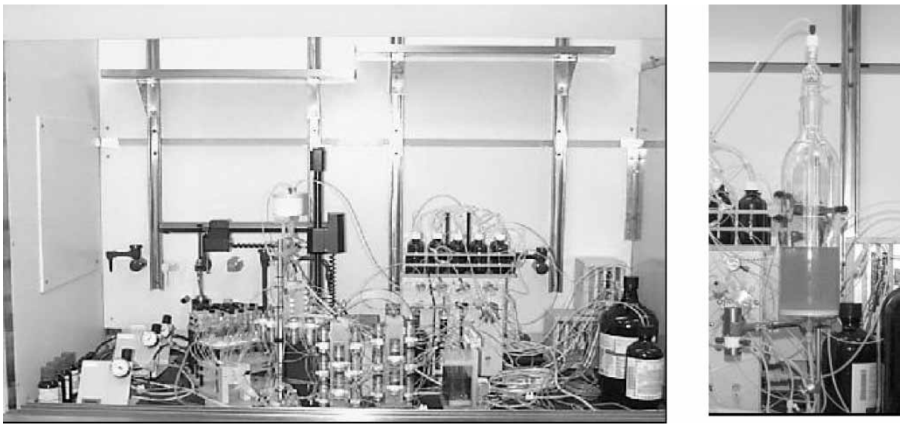

*Figure 2. (Left) Zymark prototype synthesizer; (Right) scale-up module.*

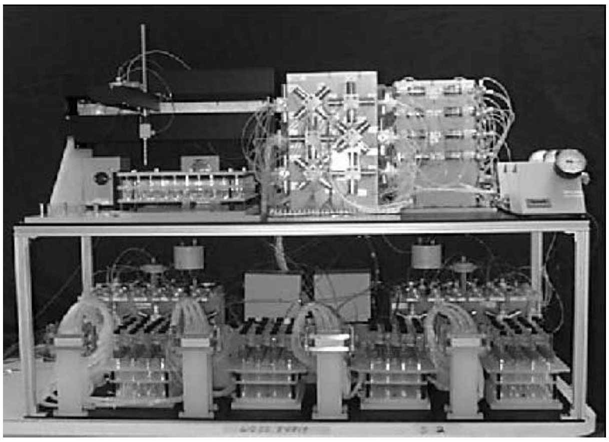

*Figure 3. Zymark production-level synthesizer.*

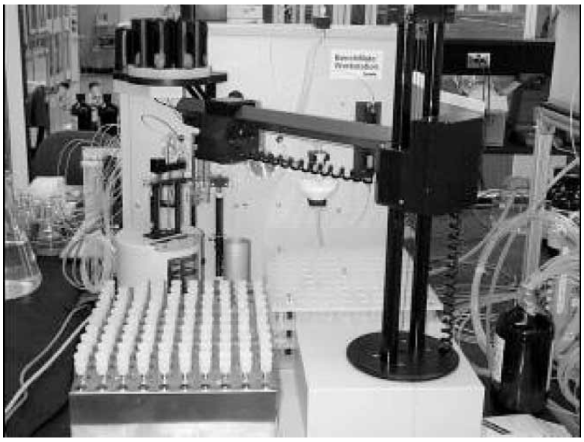

*Figure 4. BenchMate™ workstation.*

panelling, library modelling, and small library synthesis (up to 4 K). Of course, this assures that our chemistry is well in hand for the eventuality of hit re-synthesis.

### *Overview of the available automation platforms*

Many commercially available synthesis platforms lacked the flexibility sought by our group:

- Limited capability to support parallel addition of reagents, and solvents.
- Most lacked functional modularity.
- Most lacked software and hardware flexibility.

Therefore, we sought an alliance with an automation partner for the purpose of designing and building a reliable flexible synthesis platform [6]. Figure 2 shows the prototype synthesizer and the scale up module designed in collaboration with Zymark Corporation.

The features include capability for the following:

- Pooled reaction chemistries.
- Multiple programmable wash cycles.
- Simple menu-driven software, which is easily modified.
- Customized reaction vessels (not size limited) up to 1 litre capacity.

Also included in this collaboration was a production level instrument for library synthesis and hit re-synthesis (figure 3).

This synthesizer features 96 reaction vessels arranged in four blocks of 24. Each block can be individually temperature controlled over a range from −78 to +150°C, while being kept under an inert environment. Reagents may be added serially or in parallel and all wash steps are done in parallel [5]. The diversity reagent preparation for this instrument can be performed on a specially modified BenchMate™ (Zymark Corp., Hopkinton, MA, USA) workstation with racks that are compatible with the production synthesizer (figure 4).

Exploratory chemistry and development chemistry are routinely performed on one of several Argonaut Technologies Quest® 205 (larger scale 10 reaction vessels) or Quest® 210 (smaller scale 20 reaction vessels) semi-automated platforms (figure 5).

The authors have recently acquired a Myriad Discoverer 3000 Synthesizer™ (Mettler-Toledo Corp., Vernon Hills, IL, USA) to strengthen our automation capabilities for solution-phase synthesis. This platform is fully automated and has the capability of performing 24 individual reactions simultaneously. It is also suited with modified reaction vessels to operate in the solid phase chemistry mode (figure 6).

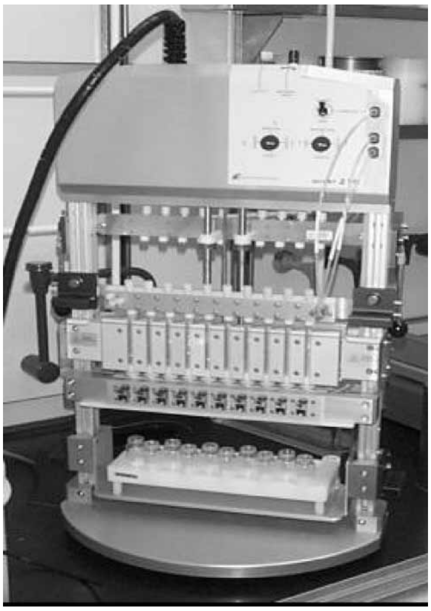

*Figure 5. Argonaut Quest® 210.*

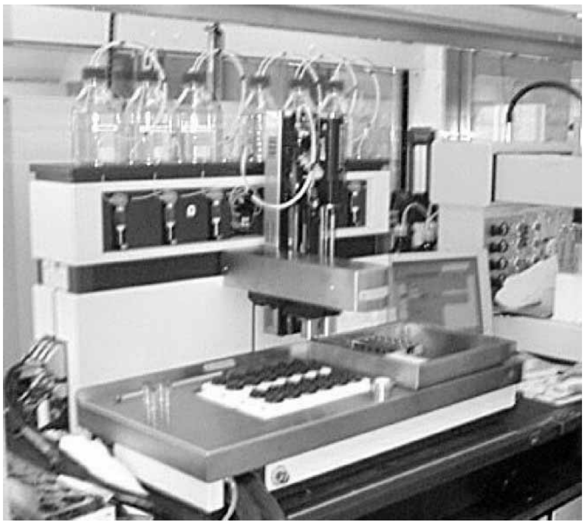

*Figure 6. Myriad Discoverer 3000 Synthesizer$^{TM}$.*

As an adjunct to the Discoverer we have also acquired a Myriad Allex$^{TM}$ liquid handler for solution phase extraction workups of products from the Discoverer (figure 7).

The main workhorse for library synthesis are a pair of Argonaut Trident$^{TM}$ automated synthesizers. Each instrument is capable of synthesizing 192 compounds/run, or 96 compounds/run on a larger scale (10 ml). This fully automated platform contains many of the features of the Zymark synthesizer described above. The design of this instrument was chemistry-driven and it is invaluable for our work. The software has gone through numerous upgrades and is extremely chemist oriented (figures 8 and 9).

The Trident® also has a companion Trident® SPS Workstation, which is manufactured by Zinsser Analytic GmbH (Germany). This workstation is compatible with

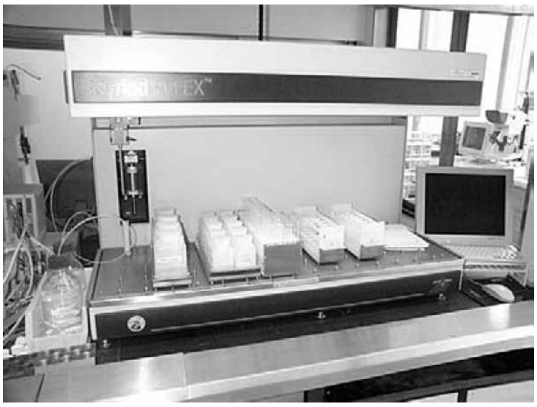

*Figure 7. Myriad Allex$^{TM}$.*

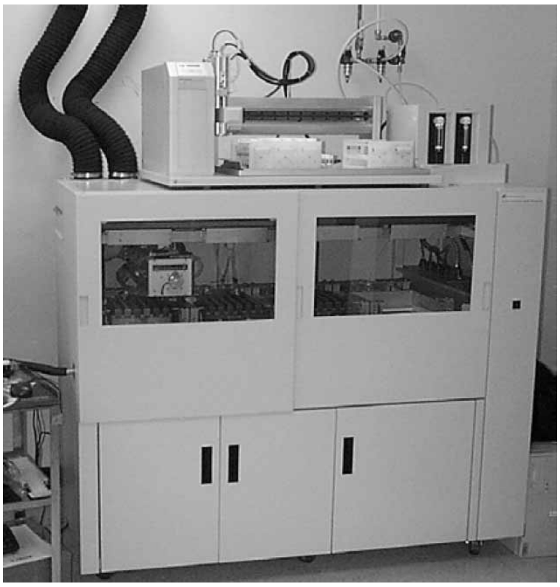

*Figure 8. Argonaut Trident®.*

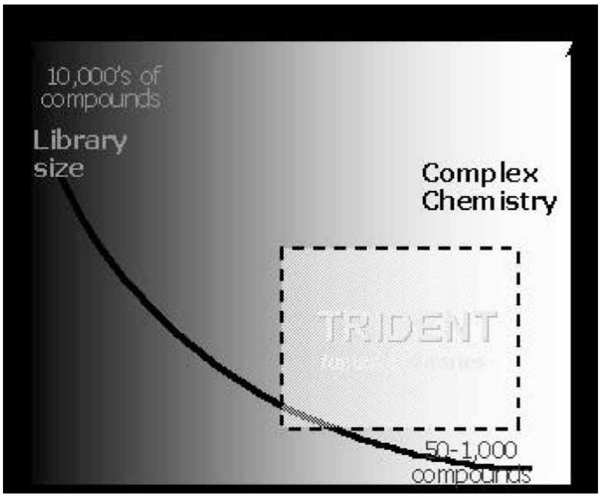

*Figure 9. For small complex molecule synthesis.*

Trident® reaction cassettes but is easily configurable to any rack which is desired. This station also features automatic resin dispensing to Trident® reaction cassette blocks (figure 10).

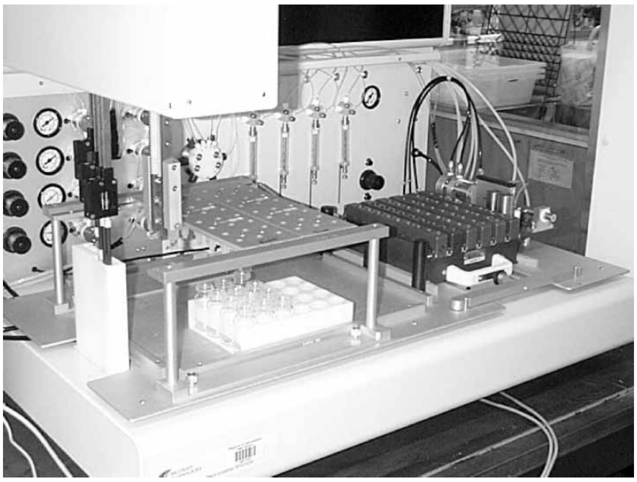

*Figure 10. Trident® SPS Workstation.*

The EMRYS™ Microwave Synthesizer (Personal Chemistry, Boston, MA, USA) is an automated serial instrument with liquid handler. The technology offers the advantage of accelerating the rate of thermally driven reactions by as much as 100-fold over normal heating techniques. One frequently observes higher chemical conversion, greater purity and minimization of undesired side reactions. This instrument has proven itself repeatedly to have great value in our synthesis group (figure 11).

With the many tools available, we find that the difficulty can often lie in choosing the right tool for the job. This requires striking a balance between becoming overly systematic as a means of limiting risk. However, this can also mean that one must balance the danger of becoming prescriptive to the exclusion of creativity.

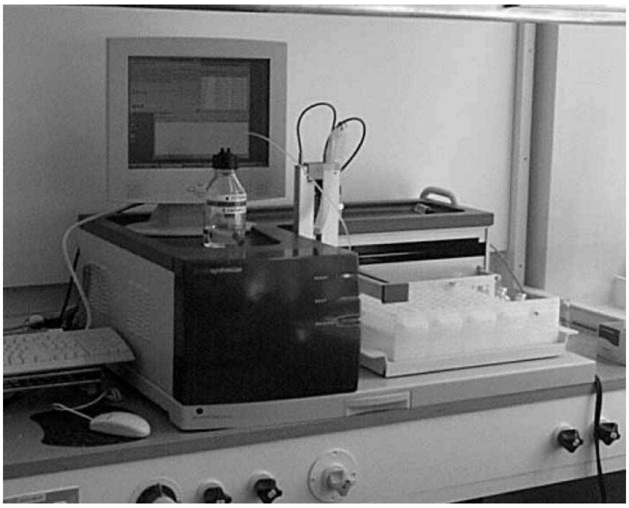

*Figure 11. EMRYS Synthesizer™.*

### References

1. Zuckerman, R. N., Kerr, J. M., Siani, M. A. and Banville, S. C., *Int. J. Peptide Protein Res.*, **40** (1992), 497.
2. Sugawara, T., Kato, S. and Okamoto, S., *J. Automation Chem.*, **16** (1994), 33.
3. DeWitt, S. H. and Czarnik, A. W., *Cur. Opinion Biotech.*, **6** (1995), 640.
4. Tan, D. S., Foley, M. A., Stockwell, B. R., Shair, M. D. and Schreiber, S. L. *J. Am. Chem. Soc.*, **121** (1999), 9073.
5. Campbell, J. B. and Wildonger, R. A. Managing compound library production by parallel synthesis on automated workstation-based systems. *ISLAR '96 Proceedings*, p. 127.
6. Wildonger, R. A. and Campbell, J. B., Progress toward the implementation of an integrated workstation-based multiple parallel solid phase synthesis system. *ISLAR '97 Proceedings*, p. 73.

